# Defined PEG smears as an alternative approach to enhance the search for crystallization conditions and crystal-quality improvement in reduced screens

**DOI:** 10.1107/S1399004715007968

**Published:** 2015-07-28

**Authors:** Apirat Chaikuad, Stefan Knapp, Frank von Delft

**Affiliations:** a Nuffield Department of Clinical Medicine, Structural Genomics Consortium, University of Oxford, Old Road Campus Research Building, Roosevelt Drive, Headington, Oxford OX3 7DQ, UK; b Institute for Pharmaceutical Chemistry, Johann Wolfgang Goethe-University, Building N240 Room 3.03, Max-von-Laue-Strasse 9, 60438 Frankfurt am Main, Germany

**Keywords:** PEG smear, crystallization screen, chemical space, protein crystallization

## Abstract

The quest for an optimal limited set of effective crystallization conditions remains a challenge in macromolecular crystallography, an issue that is complicated by the large number of chemicals which have been deemed to be suitable for promoting crystal growth. The lack of rational approaches towards the selection of successful chemical space and representative combinations has led to significant overlapping conditions, which are currently present in a multitude of commercially available crystallization screens. Here, an alternative approach to the sampling of widely used PEG precipitants is suggested through the use of PEG smears, which are mixtures of different PEGs with a requirement of either neutral or cooperatively positive effects of each component on crystal growth. Four newly defined smears were classified by molecular-weight groups and enabled the preservation of specific properties related to different polymer sizes. These smears not only allowed a wide coverage of properties of these polymers, but also reduced PEG variables, enabling greater sampling of other parameters such as buffers and additives. The efficiency of the smear-based screens was evaluated on more than 220 diverse recombinant human proteins, which overall revealed a good initial crystallization success rate of nearly 50%. In addition, in several cases successful crystallizations were only obtained using PEG smears, while various commercial screens failed to yield crystals. The defined smears therefore offer an alternative approach towards PEG sampling, which will benefit the design of crystallization screens sampling a wide chemical space of this key precipitant.

## Abstract 2

An alternative strategy for PEG sampling is suggested through the use of four newly defined PEG smears to enhance chemical space in reduced screens with a benefit towards protein crystallization.

## 1. Introduction   

Crystallization remains one of the bottleneck steps in macromolecular crystallography. This process usually involves an initial broad screening for effective cocktails known to promote crystallization. The large number of precipitants, buffers and additives currently used in crystallization experiments hinders a complete and systematic combinatorial approach in experiments that are constrained by protein availability and costs. These restrictions led to the invention of several empirical approaches, such as the sparse-matrix (Jancarik & Kim, 1991 ▸), incomplete-factorial (Carter & Carter, 1979 ▸) and grid formulations (Brzozowski & Walton, 2001 ▸; McPherson, 2001 ▸; Gorrec, 2009 ▸). Since most commercially available randomized screens are often formulated around a small set of particular favourable reagents with highly overlapping chemical properties (Gorrec, 2013 ▸), one or two of these screens should generally be sufficient to enable successful crystallization outcomes (Segelke, 2001 ▸; Newman *et al.*, 2005 ▸). However, employing these few screens may not enable the broad chemical space sampling necessary to identify suitable crystallization conditions in some cases.

Owing to its ability to promote protein–protein association (McPherson, 1976 ▸; Brzozowski & Tolley, 1994 ▸; George & Wilson, 1994 ▸; Vivarès & Bonneté, 2002 ▸; Budayova *et al.*, 1999 ▸; Tanaka & Ataka, 2002 ▸; Tanaka *et al.*, 2003 ▸; Kulkarni *et al.*, 2000 ▸), polyethylene glycol (PEG) has been widely used as one of the most effective precipitants in protein crystallization (Fazio *et al.*, 2014 ▸). Various degrees of polymerization and additional modifications generate a large variety of PEG variants with molecular weights that range from 200 to >20 000 Da. Based on the Biological Macromolecule Crystallization Database (BMCD; http://xpdb.nist.gov:8060/BMCD4/index.faces; Gilliland *et al.*, 2002 ▸), at least 20 PEGs (200, 300, 350 MME, 400, 500 DME, 550 MME, 600, 750 MME, 800, 1K, 2K, 2K MME, 3350, 4K, 5K MME, 6K, 8K, 10K, 12K and 20K) are frequently used as effective precipitants in macromolecular crystallization. It is impractical to sample all varieties of PEGs in an initial crystallization screening, because a set of additives and buffers would need to be repetitively combined with each of these different PEG variants. Therefore, a few types of this polymer are predominantly selected in commercial screens to allow the exploration of other chemicals. This approach may be justified by the notion that a reduced screen formulated around two or three PEG variants would be sufficient to identify the conditions needed for subsequent follow-up experiments (Kimber *et al.*, 2003 ▸; Gao *et al.*, 2005 ▸; Page *et al.*, 2003 ▸) such as orthogonal approaches (Kingston *et al.*, 1994 ▸) or reverse screening (Stura *et al.*, 1994 ▸). This PEG-sampling strategy hence cannot provide full coverage of PEG chemical space in the initial crystallization, leading to the use of several commercial screens in small- and medium-sized laboratories. However, a recent survey has suggested that more random screening still undersamples crystallization conditions (Gorrec, 2013 ▸).

During the development of the PACT screen, the so-called PEG smear was introduced and was made up by mixing ten different PEGs to smear out the molecular-weight range of the polymer from 200 to 10 000 Da (Newman *et al.*, 2005 ▸). A PEG smear (or any pooled set of chemicals) requires that the effects of each component towards crystallization are either neutral or positive. A benefit of the smear, which should in principle preserve the chemical properties of each included polymer, is that it would reduce the number of PEG variables while potentially maintaining a large coverage of PEG space. In preliminary macromolecular crystallization tests (Newman *et al.*, 2005 ▸), the comparable success rates of smear-based and single PEG-based screens have suggested a potential substitution of individual, single PEGs with smear precipitants in the formulation of crystallization screens. In addition, examples of successful structure determinations using the smear precipitant have also been demonstrated (Aricescu *et al.*, 2007 ▸; Koski *et al.*, 2009 ▸). Nonetheless, several studies have shown that homogeneity regarding the molecular weights and concentrations of PEGs are vital determinant factors to promote protein crystal growth (McPherson, 1976 ▸; Valjakka *et al.*, 2000 ▸; Stura *et al.*, 1994 ▸; Snell *et al.*, 2008 ▸), since their fluctuations could affect potential intermolecular interactions (Tanaka & Ataka, 2002 ▸; Kulkarni *et al.*, 2000 ▸; Budayova *et al.*, 1999 ▸). Therefore, it remains unclear whether this type of smear with huge heterogeneities and less conservation of the diverse physical properties associated with different molecular weights would be an optimal approach to sample this polymer.

In this study, we defined four new types of PEG smears formulated from 11 PEGs based on their molecular-weight classes as an extended approach to explore the use of this precipitant in macromolecular crystallization. These included low-, medium-, high- and broad-molecular-weight smears, in which the latter was equivalent to the previously reported combination (Newman *et al.*, 2005 ▸). An initial systematic test on the use of these precipitants for the crystallization of a set of 32 problematic proteins suggested that the combinatorial use of all four PEG smears enhanced crystallization efficacy by this wide sampling of PEGs. We then formulated a smear-based randomized screen by the introduction of a multiple-additive strategy. This screen was tested for crystallization potency using a set of 191 proteins from the current projects at the Structural Genomics Consortium (SGC). Interestingly, the smear-based screen showed nearly similar success rates to the success rate of the four commercial screens used together. In addition, of particular significance was the ability of the smear-based screen to promote the crystal growth of several proteins that were resistant to crystallization using the commercial sets.

## 2. Materials and methods   

### 2.1. Materials   

**Figure 1:**
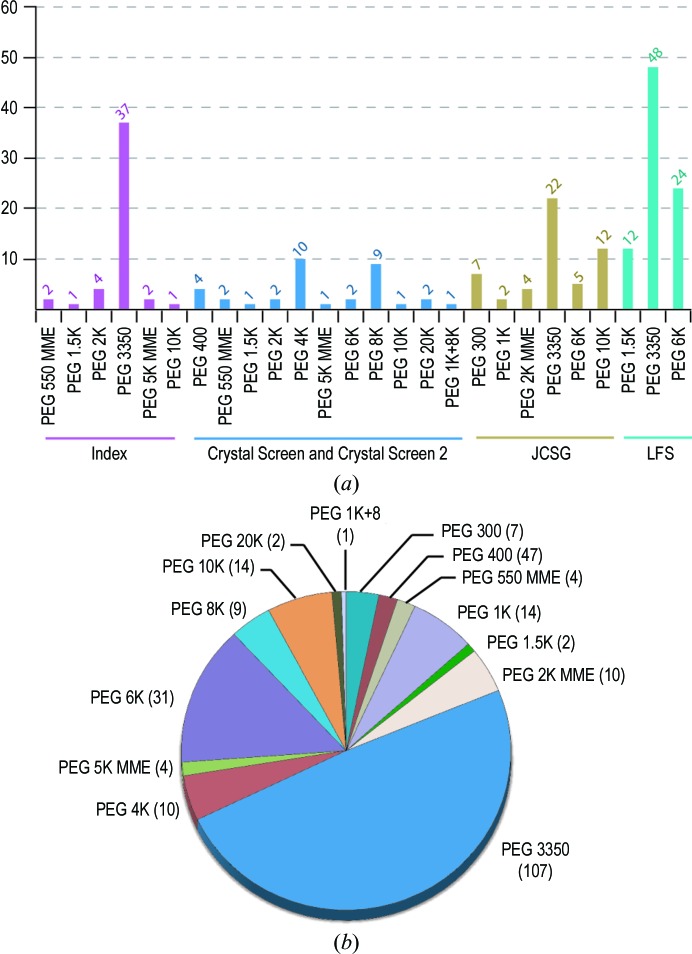
Analysis of the usage of single PEGs in the four primary screens. (*a*) Lists of PEGs and their frequency of use in the benchmarking set of four widely used commercially relevant screens routinely employed during initial crystallization at the SGC. (*b*) Pie chart demonstrating the proportion of each PEG used in the total 218 PEG-based cocktails of the four commercially relevant primary screens, revealing a highly biased sampling towards some particular PEG variants.

11 PEGs, including PEG 400, 550 MME, 600, 1K, 2K, 3350, 4K, 5K MME, 6K, 8K and 10K, which were available in-house and are commonly used in most commercial screens (Fig. 1 ▸), were chosen in this study to provide a molecular-weight (MW) coverage ranging from 400 to 10 000 Da. Stock solutions were purchased from Fluka, NeXtal and Rigaku Reagents. Typical concentrations were at 50%(*w*/*v*), except for some low-molecular-weight types, which were diluted to 50%(*v*/*v*) prior to use. All other chemicals, including buffers, organic and salt solutions, were of analytical grade and were purchased from Rigaku Reagents and Sigma.

### 2.2. Definition and preparation of PEG smears   

The PEGs were divided into three classes: low molecular weight (≤1 kDa), medium molecular weight (>1–5 kDa) and high molecular weight (≥6 kDa). The PEG smears were made by mixing PEG stocks (50% concentration) at an equal volume to give the smear stocks and an overall concentration of 50%. Four smears were created: (i) low molecular weight (LMW; PEG 400, 550 MME, 600 and 1K), (ii) medium molecular weight (MMW; PEG 2K, 3350, 4K and 5K MME), (iii) high molecular weight (HMW; PEG 6K, 8K and 10K) and (iv) broad molecular weight (BMW; PEG 400, 550 MME, 600, 1K, 2K, 3350, 4K, 5K MME, 6K, 8K and 10K). The formulation of the BMW smear was similar to that of the smear used previously (Newman *et al.*, 2005 ▸).

### 2.3. Test proteins and crystallization experiments   

Recombinant human proteins from current projects at the SGC were used as test proteins. In order to evaluate the efficacies of the four smears in crystallization, two PEG smear-based screens were formulated. All crystallization experiments were performed in 96-well, three-subwell SWISSCI plates (SWISSCI AG) using the sitting-drop vapour-diffusion method at 277 and/or 293 K. Reservoirs of 20 µl were used for equilibration against 150 nl fixed-volume crystallization drops, which were prepared at 2:1, 1:1 and 1:2 protein:reservoir volume ratios (Luft *et al.*, 2007 ▸). Crystallization drops were imaged regularly over a period of two months using an automatic inspection system (Rigaku). Parallel experiments with four primary screens, JCSG+ (hereafter referred to as JCSG), PACT equivalent (hereafter referred to as LFS), Hampton Research Crystal Screen and Crystal Screen 2, and Hampton Research Index, were also performed under identical experimental parameters such as protein concentrations, protein buffers, drop ratios and temperatures.

## 3. Results and discussion   

### 3.1. Initial PEG smear-based screen and preliminary crystallization test   

**Figure 2:**
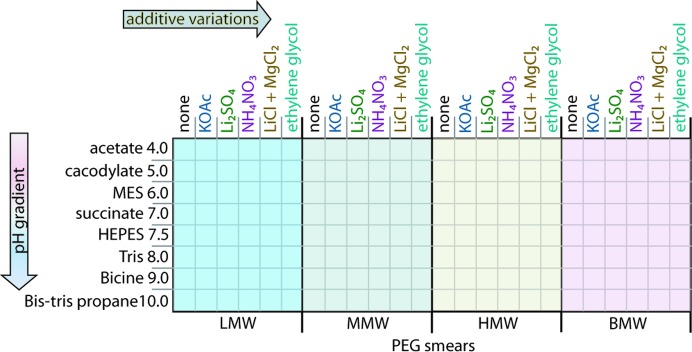
The systematic design of the smear-based BCS1. The illustration demonstrates the complete factorial systematic design of the initial PEG smear-based screen BCS1. The buffers were used at a concentration of 0.1 *M* and the additives were (i) potassium acetate (KOAc), (ii) lithium sulfate (Li_2_SO_4_), (iii) ammonium nitrate (NH_4_NO_3_), (iv) lithium chloride and magnesium chloride mixture (LiCl + MgCl_2_) and (v) ethylene glycol, all at 0.2 *M* concentration apart from ethylene glycol, which was at 10%(*v*/*v*). The PEG smears used are low molecular weight (LMW), medium molecular weight (MMW), high molecular weight (HMW) and broad molecular weight (BMW).

Initially, proteins that had shown themselves to be resistant to crystallization using our four primary screens were the primary focus of the experiment. The strategy was to develop an alternative in-house screen with a more diverse chemical space, which would also provide further information regarding the protein-precipitation behaviour in the drops and would hopefully increase the crystallization success rate. Based on our observation that PEG space was poorly covered in our four primary sparse-matrix screens (Fig. 1 ▸), we considered expanding the use of diverse PEG precipitants during initial crystallization. This led to the development of four PEG smears with an equal sampling of the polymer. The four PEG smears allowed a complete-factorial orthogonal array approach (Kingston *et al.*, 1994 ▸) for the design of an initial screen. This screen, hereafter referred to as Basic ChemSpace 1 (BCS1), comprised a total of 192 cocktails and was based around the four smears at a fixed concentration of 22.5%, eight buffer systems covering the pH range 4–10 and five additives known to promote crystallization (Snell *et al.*, 2008 ▸) that are, however, rarely or never sampled in the four primary screens (Fig. 2 ▸).

The efficacy of this initial smear-based screen was tested on a set of 32 recombinant human proteins, 25 of which failed to crystallize when tested with the four primary screens; the seven remaining targets had not yielded diffraction-quality crystals. Unexpectedly, the BCS1 screen led to crystallization hits for 15 of these proteins. The crystallized proteins were from diverse protein families including kinases (five), phosphatases (one), dehydrogenases (four) and others (five). It is noteworthy that eight of these proteins had never crystallized previously. In addition, most of the crystals of these test proteins obtained from the BCS1 screen resulted in successful structure determinations (see also §[Sec sec3.4]3.4 for PGAM5, MMAA and UGDH).

### 3.2. Comparison of the precipitant efficiencies of the four newly defined PEG smears   

**Figure 3:**
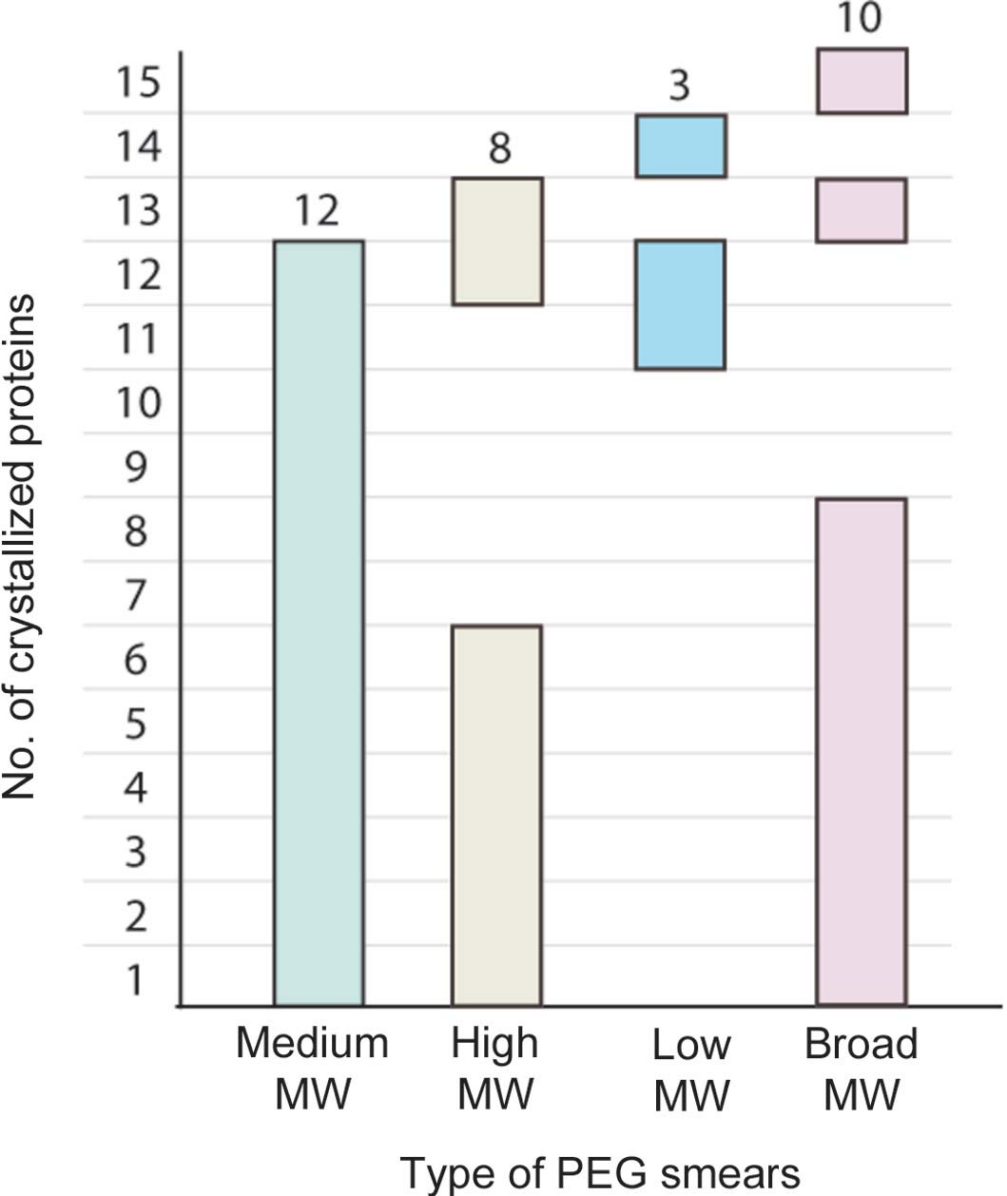
Analysis of the precipitant potencies of the four smears from the BCS1 screen. Graph showing the number of crystal hits for each smear, investigating 15 proteins crystallized in the systematic BCS1 screen. In most cases crystal hits were observed in more than one type of PEG smear; however, some proteins required a particular smear for successful crystal growth.

The excellent crystallization success rates of the initial smear-based systematic BCS1 screen allowed the evaluation of the precipitant efficiencies of each smear, which revealed that the MMW smear was the precipitant with the highest success rate, enabling the crystallization of 12 proteins (80% of the 15 crystallized proteins), while the LMW smear showed the lowest precipitant efficacy, with only an ∼20% success rate (Fig. 3 ▸). Surprisingly, despite containing all 11 PEGs, the BMW smear yielded crystal hits for only ten proteins, and its crystallization efficacy did not match the combined success rates of the other three smears (14 proteins). It was also observed that the BMW smear was not suitable as a precipitant for five proteins, the crystallization of which was only achieved using particular types of smears (Fig. 3 ▸). This was also suggested by the three proteins that crystallized exclusively in the LMW smear but not in the BMW smear. Nonetheless, a unique crystallization success for one protein that crystallized only in the BMW smear also suggested possible specific crystallization properties of the BMW smear that may extend beyond the other three smears. The results overall suggested that each smear potentially had unique precipitant properties, which may be related to their molecular weights, different levels of heterogeneity and different concentrations of effective components. However, it should also be noted that these statistics were based on a single experiment. In addition, owing to highly similar chemistry, a repetition of the BCS1 screen with the application of cross-seeding from effective smear-based conditions might potentially enable crystal growth in the counterpart conditions containing other smears which did not initially demonstrate crystallization potency.

**Figure 4:**
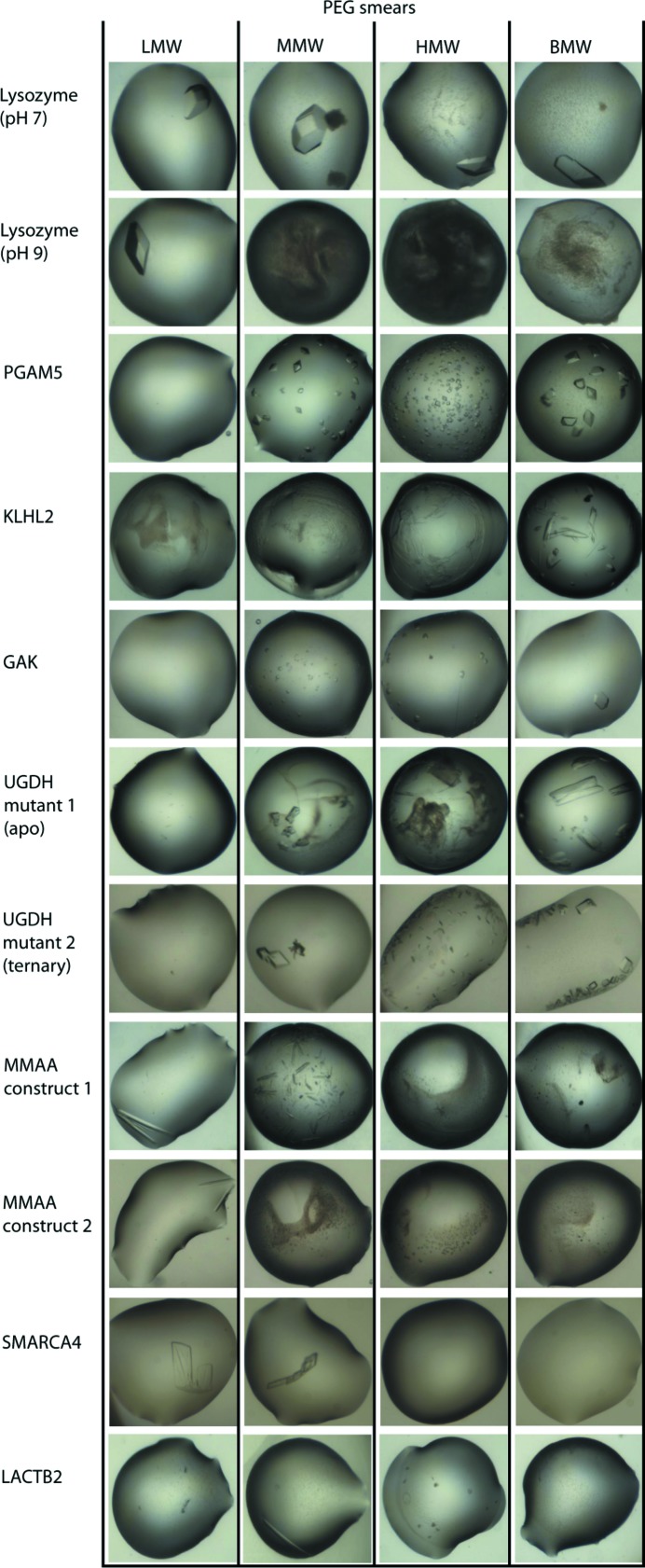
Comparison of crystal morphology across different smear precipitants in BCS1. The representative set of proteins demonstrated the effect of different smears on protein behaviour in the crystallization drops. Variations in crystal quality and morphology across different effective smear precipitants are also evident.

In addition to the efficiency of the smears in promoting crystallization, the quality of the protein crystals was also an important criterion for the validation of effective precipitants in this study. We then performed comparisons of morphology (size and regularity in three-dimensional shape) and, in some cases, the diffraction quality of the crystals of the same proteins obtained from different smears. In nearly all cases we observed high variation in crystal quality across the different types of smears (Fig. 4 ▸). For example, UGDH mutant crystals could only be obtained in the MMW, HMW and BMW smears, but only those grown in the MMW and BMW precipitants had improved morphology and diffraction quality. In addition, both LMW and MMW smears enabled MMAA crystal growth, but only the former yielded larger, single crystals with better diffraction quality than observed in the other smear. Furthermore, PGAM5 crystallized readily when using the MMW, HMW and BMW smears, but only the MMW and BMW smears yielded diffracting crystals. In some cases, however, the different classes of smears did not lead to significant differences in crystal quality, as observed in the case of the bromodomain of SMARCA4, for which crystals obtained from both LMW and MMW smears exhibited a similar diffraction quality despite having slightly different morphologies.

Overall, this preliminary crystallization experiment showed that each of the smears had unique crystallization properties and that a broad smear with high heterogeneity may not serve as a replacement for other PEG combinations. Nonetheless, the only partial overlap of precipitant efficiencies suggested that a combinatorial use of PEG in the form of these four smears generated an efficient screening set for initial crystallization trials maximizing PEG diversity and potency.

### 3.3. A 96-formatted smear-based screen and crystallization test   

After the preliminary systematic test, we believed that each of the four smears would bring unique properties to protein crystallization, and we therefore implemented a more convenient 96-well PEG smear-based crystallization screen (hereafter referred to as BCS2). We aimed to generate a screen with a wide PEG and chemical coverage in a limited experimental space, which would benefit our current projects. Therefore, instead of replacing single PEGs with their equivalent smears in the four primary screens for direct comparison between the effects of the smears *versus* single PEGs, a new set of cocktails formulated around these four precipitants was generated. The screen was divided into two parts, the design of which was based either on systematic or randomized principles and each of which is described below.

#### 3.3.1. A systematic set of simple conditions   

The first part of the screen explored only two components, PEGs and buffers, and contained 24 cocktails generated from a complete factorial combination between the four smears and six different buffer systems with pH values ranging from 4.5 to 9.5 (Table 1 ▸, set 1). The use of the smears enabled a complete assessment of the effects of the different classes of polymer under various pH conditions, which would require a larger experimental space with single PEGs. We also observed that this set of simple cocktails had the potential to promote crystal growth in several cases, including PGAM5, SMARCA4 (Filippakopoulos *et al.*, 2012 ▸), UGDH (Egger *et al.*, 2011 ▸, 2012 ▸), BAZ2B (Ferguson *et al.*, 2013 ▸), p38α–TAB1 (De Nicola *et al.*, 2013 ▸) and PCAF (see §[Sec sec3.4]3.4).

#### 3.3.2. Randomized set of the screen   

The second part of the screening conditions aimed to combine multiple factors, such as salts and additives, in addition to precipitants and buffers, and hence employed a randomization approach to formulate 72 cocktails (Table 1 ▸, set 2). Often in such a limited experimental space, expansion of chemical space comes at the expense of reduced PEG space or *vice versa*, owing to replication of conditions that differ only in different PEGs. We were able to overcome this limitation using the PEG-smear approach. We also implemented the use of multiple salts/additives in some cocktails in an attempt to further broaden chemical space. This approach may potentially offer a random search for an effective reagent from multiple components similar to the silver bullet method (McPherson & Cudney, 2006 ▸) or screens for multiple small-molecule/additive components that may be required for crystal growth.

#### 3.3.3. Crystallization efficiency   

**Figure 5:**
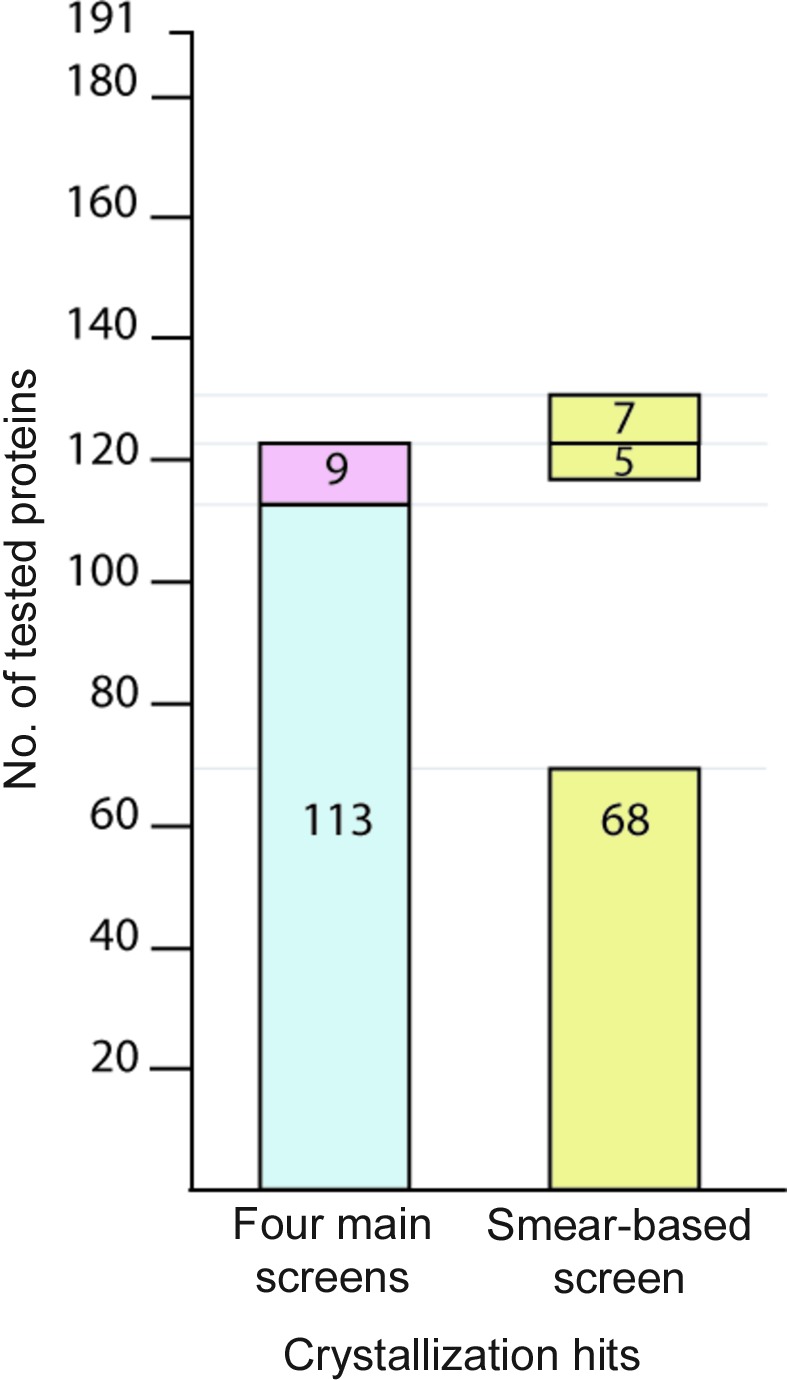
Comparison between the success rates of the four primary screens and the smear-based BCS2 screen on crystallization tests of 191 human recombinant proteins. The graph demonstrates the numbers of proteins crystallized in the four primary screens and the BCS2 screen. For the four primary screens, the blue region indicates proteins that crystallized in PEG-based conditions, while the pink region represents proteins that only crystallized in salt-based conditions.

**Figure 6:**
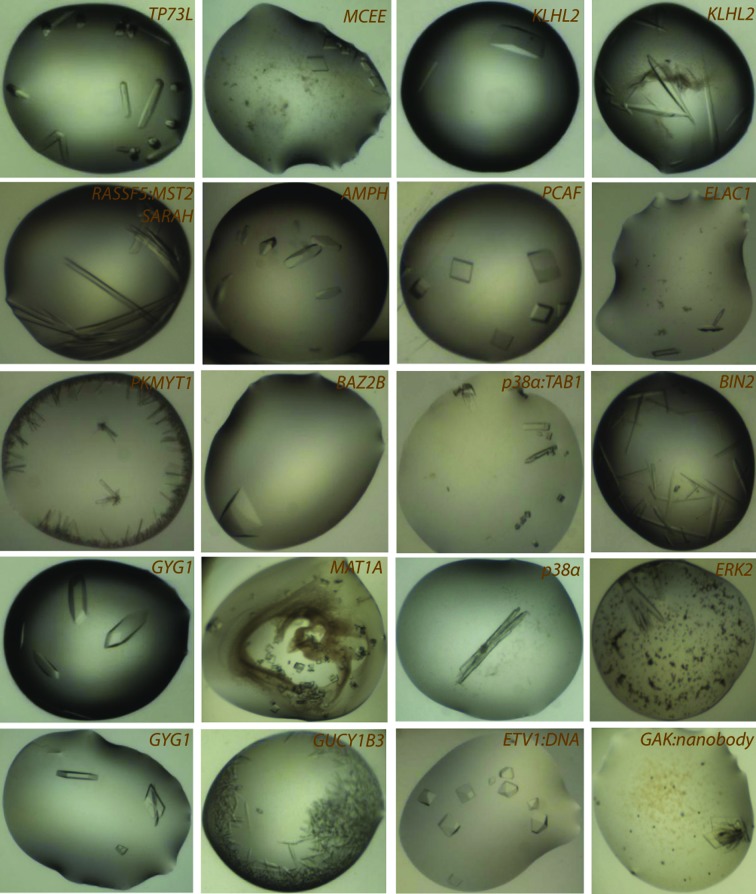
Gallery of example crystals obtained from the smear-based BCS2 screen.

The performance of the BCS2 screen was tested using a set of 191 recombinant human proteins and was compared with the combined success rate of our four primary screens. The smear-based screen demonstrated an ability to yield initial crystallization hits for 80 proteins, a 42% success rate. By comparison, this was somewhat lower than the combined success rate of the four primary screens of ∼64% (122 proteins), or 59% (113 proteins) when considering only PEG-based conditions (Fig. 5 ▸). Nonetheless, crystallization hits for 80 proteins, including seven proteins which failed to crystallize in the four primary screens and five proteins for which crystals were only obtained in salt-based conditions, was a remarkable success rate considering the limited set of crystallization cocktails (Table 2 ▸ and Fig. 6 ▸). We also observed the usefulness of the multiple salts/additives approach for crystallization of some proteins. Details of some cases such as the ETV1–DNA complex, CDKL5 and RASSF3 are discussed in §[Sec sec3.4]3.4.

**Figure 7:**
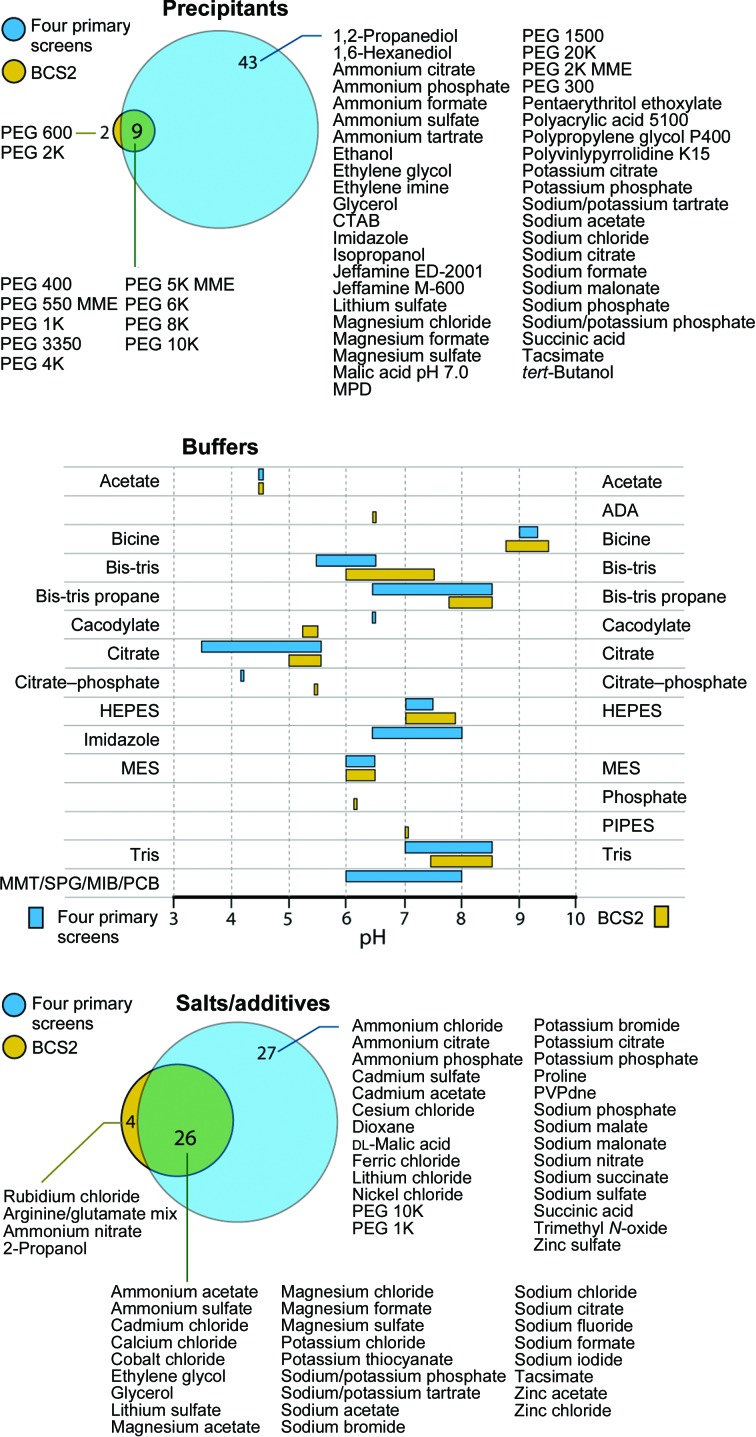
Comparison of chemical space coverage between the BCS2 and the four primary screens.

Since the BCS2 screen was not formulated by the substitution of single PEGs in the primary screens with the equivalent smears, a direct comparison of crystallization success rates was difficult. The gap between the success rate of the BCS2 screen and that of the four primary screens could be owing to the different scale of the experiments, since the BCS2 screen contained only 96 cocktails compared with the 384 conditions present in the four primary screens. In addition, the chemical space analyses further demonstrated that despite comprising 11 types of PEGs, 14 buffers with pH ranging from 4.5 to 9.5 and 30 additives, there were still a number of chemicals that were not sampled in the BCS2 screen but were present in the four primary screens (Fig. 7 ▸). Indeed, analyses of the chemistry of the crystallization conditions of 49 proteins which crystallized only in the four primary screens revealed that most of these missing components in the BCS2 screen were essential factors that promoted crystal growth in these cases. Therefore, a cause of ineffectiveness of the BCS2 screen towards these 49 proteins could be owing to the lack of such chemical space coverage, which included (i) buffers such as citrate, tris, bis-tris and bis-tris propane at different pH values (15 cases), (ii) salt/additives such as ammonium citrate, nickel chloride, potassium citrate, sodium malonate, sodium nitrate, sodium sulfate and succinic acid (seven cases), (iii) correct combinations of salt/additives and buffers (ten cases), (iv) PEGs such as PEG 300 and 20K (two cases), (v) a preference towards single, particular PEGs (11 cases) and (vi) a preference towards salt or organic precipitants (four cases). Considering the first three factors, it is possible that an implementation of another independent smear-based screen in the current BCS2 screen with extended chemical space coverage could feasibly bridge the gap in the observed success rates between the smear-based screens and the four primary screens. Overall, the results suggested that the newly defined smears, which potentially enable lowering PEG variables and biases, may provide benefit towards a formulation of the initial crystallization screen with an enhancement of both PEG and chemical space in a limited set of experiment.

### 3.4. Case studies   

#### 3.4.1. Novel conditions for crystal growth   

**Figure 8:**
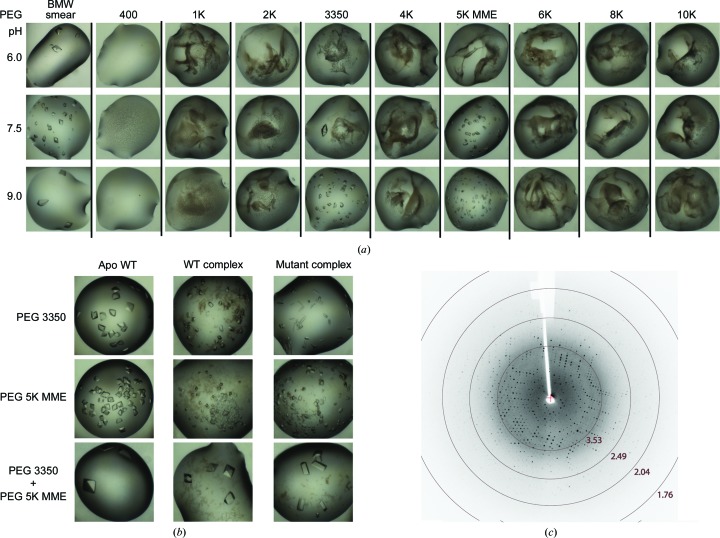
Crystallization of PGAM5 using PEG smears. (*a*) After MMW and BMW smears were identified as effective precipitants, a deconvoluted screen with single PEGs was performed which identified two PEGs, PEG 3350 and PEG 5K MME, as the effective component in the smears that promoted crystal growth. The specific smear obtained by mixing these two PEGs shows a greater crystallization efficacy towards crystal growth of the wild-type (WT) protein, the wild type–ligand complex and the mutant compared with the two single PEGs, as demonstrated by changes in crystal morphology (*b*) and the quality and resolution of the collected X-ray diffraction data (*c*).

*Phosphoglycerate mutase 5*. Human phosphoglycerate mutase 5 (PGAM5) is a mitochondrial protein that belongs to the phosphoglycerate mutase (PGAM) family and possesses Ser/Thr phosphatase activity (Takeda *et al.*, 2009 ▸). Failure to crystallize the catalytic domain using the four primary screens led to subsequent trials with the smear-based screens, which unexpectedly yielded a number of hits in conditions containing MMW, HMW and BMW smears in the pH range 6.5–8.0. Only the crystals growing in the MMW-based and BMW-based cocktails diffracted; however, there was no improvement of diffraction beyond 3 Å resolution during several rounds of typical optimization by varying the smear concentration and pH ranges. We hypothesized that this could be owing to the heterogeneity of the precipitant, and therefore attempted to deconvolute the effects of the individual PEG compositions present in the smears. We found that indeed only PEG 3350 and PEG 5K MME promoted crystal growth (Fig. 8 ▸ *a*). Although we were able to improve the crystal quality slightly, the limitation in diffraction resolution still remained, potentially owing to the small size of the crystals caused by the large number of nucleation sites in the drops (Fig. 8 ▸ *b*). We then mixed the two single PEGs to create a specific smear, which interestingly was the most effective precipitant, reliably producing large crystals that diffracted to high resolution (Figs. 8 ▸ *b* and 8 ▸ *c*). In addition, this specific smear was also suitable for the crystallization of both wild-type and mutant ligand complexes (PDB entries 3mxo and 3o0t; Structural Genomics Consortium, unpublished work; Fig. 8 ▸ *b*).

*ETV1 in complex with DNA*. The DNA-binding domain of ETS translocation variant 1 (ETV1) crystallized readily in various conditions, yet obtaining crystals of the complex of ETV1 with DNA proved to be difficult, with no convincing hits identified from our four primary screens. A smear-based condition containing the LMW smear, 0.1 *M* Tris pH 8.5, 0.2 *M* NaCl, 5% glycerol yielded diffraction-quality crystals of the complex, resulting in structure determination at 2.9 Å resolution (PDB entry 4bnc; Cooper *et al.*, 2015 ▸). Analysis of the composition of this effective condition revealed that similar chemicals, low MW PEG, basic pH and monovalent salts, were used in several cocktails in the primary screens, albeit with an unsuccessful crystallization outcome. This suggested an inadequate sampling of PEG properties with correct combinations in the primary screens and the benefit of the PEG smears and/or multiple salts/additives.

#### 3.4.2. Identification of PEG smear conditions improving diffraction quality   

**Figure 9:**
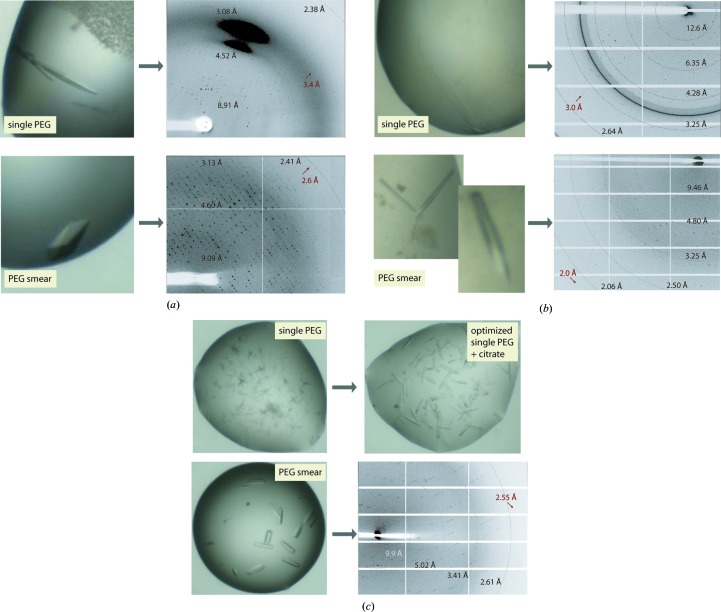
Improvement of the diffraction quality of crystals growing in the smear-based screen. Comparison between the morphology and the X-ray diffraction quality for crystals of MMAA (*a*) and CDKL5 (*b*) obtained from the single PEG-based and smear-based conditions. (*c*) The quality of RASSF3 crystals growing in the effective smear-based condition edges those of the crystals from the single PEG condition with or without the incorporation of citrate buffer found as a composition in the smear-based cocktail. See text for details.

*MMAA*. Crystals of mitochondrial methylmalonic aciduria type A (MMAA) grew readily in a number of conditions present in the four primary screens which contained either PEG 6K or PEG 10K as a precipitant, bis-tris buffer pH 6–7 and divalent or monovalent chloride salts. However, no improvement in diffraction beyond 3.5 Å resolution was obtained after an elaborate optimization strategy (Fig. 9 ▸ *a*). Trials with the smear-based screens revealed LMW and MMW smears as effective precipitants, with the former yielding initial crystals that diffracted X-rays to ∼3.2 Å resolution. A simple optimization of the best condition, containing the LMW smear supplemented with ammonium nitrate and buffered to pH 5.0 with cacodylate, led to crystals that diffracted to 2.6 Å resolution (Fig. 9 ▸ *a*; Froese *et al.*, 2010 ▸). Interestingly, the structure revealed interactions of a number of low-molecular-weight PEG molecules at the protein–protein interface, suggesting stabilizing and nucleating properties of the low-molecular-weight PEG components (PDB entry 2www).

*CDKL5*. A mixture of PEG 20K and MES buffer pH 5–6 was identified as the most effective condition for the crystallization of cyclin-dependent kinase-like 5 (CDKL5) from the four primary screens. However, the diffraction quality of the thin, plate-like, monoclinic crystals remained poor, with a resolution limit of 3.0 Å (Fig. 9 ▸ *b*). Crystallization hits were also identified from several cocktails of the smear-based screen, which included (i) MMW smear, 0.1 *M* Tris pH 8.0, 0.075 *M* sodium acetate, 0.15 *M* NaCl; (ii) MMW smear, 0.1 *M* bis-tris propane pH 8.0, 0.01 *M* CoCl_2_, 0.2 *M* MgCl_2_, 2% glycerol; (iii) MMW smear, 0.1 *M* bicine pH 9.3; (iv) MMW smear, 0.1 *M* HEPES pH 7.2, 7% Tacsimate; and (v) HMW smear, 0.1 *M* Tris pH 8.0, 0.2 *M* MgCl_2_, 10% glycerol. Comparison of the previous single PEG-based and smear-based conditions suggested that the MMW class of PEGs and basic pH were the main differences from the initial condition identified from the primary screens. Interestingly, these smear-based cocktails changed the morphology of the crystals to a rod-like shape with an orthorhombic Bravais lattice and considerably improved the diffraction to 2 Å resolution (PDB entry 4bgq; Fig. 9 ▸ *b*; Structural Genomics Consortiun, unpublished work). This case supports the utility of the use of the PEG smears containing multiple salts/additives to sample a larger chemical space in an initial crystallization.

*RASSF3*. Two crystallization hits were identified for the RAS-associating domain of RASSF3, which consisted of (i) PEG 4K, 0.2 *M* ammonium sulfate and (ii) MMW smear, 0.1 *M* citrate pH 5.5, 0.1 *M* ammonium sulfate, 0.05 *M* MgSO_4_. The morphologies of the crystals obtained from these two conditions were different: needles in the PEG 4K-containing condition and a rod-like form in the MMW smear (Fig. 9 ▸ *c*). After several rounds of optimization based on the PEG 4K-based condition, the diffraction quality remained poor despite an improvement in the morphology upon supplementation with citrate buffer, one of the main different components in the smear-based condition. For comparison, the rod-like crystals obtained from the smear-based condition produced X-ray diffraction to ∼2.6 Å resolution (Fig. 9 ▸ *c*). This example further demonstrated the use of PEG smears as a defined precipitant to improve crystal quality.

#### 3.4.3. Enhanced chemical space facilitating a search for alternative crystal forms   

*UGDH*. The two initial ternary structures of UDP-glucose dehydro­genase (UGDH) in rhombohedral and monoclinic lattices revealed both domains of the hexameric enzyme in a closed state (Egger *et al.*, 2011 ▸). Attempts to crystallize the protein in an open-state apo form or a form that bound only the cofactor or substrate failed not only in the previously used conditions but also in the four primary screens. Interestingly, these forms of the enzyme crystallized readily in two smear-based conditions, both containing the BMW smear mixed either with MES pH 6.0 or HEPES pH 7.5 and 5% glycerol, inducing different packing of the protein in two new orthorhombic lattices showing two slightly different open states (PDB entries 3itk and 3khu; Egger *et al.*, 2011 ▸, 2012 ▸).

*p38α in complex with the TAB1 peptide*. TAB1 is an adaptor protein that has been reported to bind to and induce the autophosphorylation of MAP kinase p38α (Ge *et al.*, 2002 ▸). Crystals of p38α in complex with the TAB1 peptide grew readily in several conditions, including those in the smear-based screens, but they all belonged to a tetragonal Bravais system (PDB entries 4lop and 4loq). Although the structure provided some insights into the conformational switch of the activation segment, the structural analysis was complicated by an unusual dimeric assembly and potential crystal-packing bias involving contacts mediated by the activation segment. The search for an alternative crystal form identified the smear-based condition containing the MMW smear and 0.1 *M* MES pH 6.5, which promoted the growth of monoclinic crystals (PDB entry 4loo). The monomeric complex observed in this crystal form captured the physiological state of the protein and hence allowed structural interpretation of the mechanism of the TAB1-induced autophosphorylation with no ambiguity (De Nicola *et al.*, 2013 ▸).

*ERK2 in complex with a specific VTX-11e inhibitor*. Wild-type ERK2 kinase in complex with inhibitors can be readily crystallized in various conditions, including some of the smear-based cocktails. The crystals typically belonged to either an orthorhombic or a monoclinic space group. However, our attempts to crystallize the complex with the specific inhibitor VTX-11e failed in the previously known conditions and the four primary screens. Interestingly, crystals of this complex were obtained in a smear-based condition containing the MMW smear, cacodylate pH 5.5 and ammonium sulfate, which induced a new packing in a hexagonal space group (PDB entry 4qte; Chaikuad *et al.*, 2014 ▸). These cases demonstrated the effectiveness of the smear-based screen in identifying alternative crystallization conditions with different packing and space groups in a limited experimental space.

## 4. Conclusion   

The lack of success in identifying crystallization conditions for several proteins encouraged us to analyse the chemical space of the standard primary screens routinely employed in our initial screening process, revealing significant overlap in the chemistry of screening conditions and insufficient sampling of the large diversity of PEGs that are commercially available. Since a comprehensive and systematic sampling of PEG variants would require large screening panels, we utilized the PEG-smear strategy and developed and analysed four newly defined types of PEG smears. We observed good crystallization success rates using proteins that we had been unable to crystallize using single PEG screening sets. The high crystallization efficacy of all four smears compared with that of the previously described broad-range smear alone suggested an improved approach in the utilization of these mixtures in crystallization. In addition, the reasonable crystallization success rate of the reduced smear-based screens towards more than 220 human recombinant proteins demonstrated the benefits of these four defined smears, which not only provided wide PEG coverage but allowed a greater sampling of other chemicals in a limited set of cocktails through a lower number of PEG variables. In several cases the PEG smears not only provided unique chemistry but yielded crystals with improved diffraction quality and with alternative crystal packing. The utility of the defined smears could offer an alternative approach towards PEG sampling in initial crystallization under limited experimental space.

## Figures and Tables

**Table:** Set 1.

No.	PEG smear	%	Buffer (0.1*M*)	pH
**1**	LMW	30.0	Acetate	4.5
**2**	LMW	30.0	Citrate/phosphate	5.5
**3**	LMW	30.0	MES	6.5
**4**	LMW	30.0	HEPES	7.5
**5**	LMW	30.0	Tris	8.5
**6**	LMW	30.0	Bicine	9.5
**7**	MMW	25.0	Acetate	4.5
**8**	MMW	25.0	Citrate/phosphate	5.5
**9**	MMW	25.0	MES	6.5
**10**	MMW	25.0	HEPES	7.5
**11**	MMW	25.0	Tris	8.5
**12**	MMW	25.0	Bicine	9.5
**13**	HMW	20.0	Acetate	4.5
**14**	HMW	20.0	Citrate/phosphate	5.5
**15**	HMW	20.0	MES	6.5
**16**	HMW	20.0	HEPES	7.5
**17**	HMW	20.0	Tris	8.5
**18**	HMW	20.0	Bicine	9.5
**19**	BMW	20.0	Acetate	4.5
**20**	BMW	20.0	Citrate/phosphate	5.5
**21**	BMW	20.0	MES	6.5
**22**	BMW	20.0	HEPES	7.5
**23**	BMW	20.0	Tris	8.5
**24**	BMW	20.0	Bicine	9.5

**Table:** Set 2.

No.	PEG smear	%	Buffer, pH (0.1*M*)	Additive 1	Additive 2
**25**	LMW	35.0			
**26**	LMW	28.0	Acetate, 4.6	0.2*M* ammonium acetate	5%(*v*/*v*) ethylene glycol
**27**	MMW	28.0		0.15*M* NaCl	
**28**	MMW	25.0	Cacodylate, 5.5	0.2*M* ammonium sulfate	
**29**	MMW	25.0	Citrate, 5.5	0.1*M* Na/K phosphate	0.1*M* RbCl
**30**	HMW	22.5		0.2*M* KCl	
**31**	HMW	15.0	Citrate, 5.0	0.15*M* ammonium acetate	
**32**	BMW	25.0		0.05*M* arginine/glutamate mix	5%(*v*/*v*) glycerol
**33**	BMW	20.0	Citrate, 5.6	0.15*M* Mg acetate	
**34**	BMW	25.0	Acetate, 4.6	0.2*M* ammonium sulfate	
**35**	LMW	22.5	MES, 6.0	0.2*M* Na/K tartrate	
**36**	MMW	22.5	PIPES, 7.0	0.1*M* CaCl_2_	0.1*M* MgCl_2_
**37**	LMW	22.5	Cacodylate, 5.3	0.2*M* ammonium nitrate	
**38**	LMW	22.5	MES, 6.5	10%(*v*/*v*) isopropanol	
**39**	MMW	20.0	MES, 6.0	0.2*M* ammonium nitrate	5%(*v*/*v*) glycerol
**40**	MMW	20.0	Phosphate, 6.2	0.2*M* Na formate	10%(*v*/*v*) glycerol
**41**	MMW	30.0	ADA, 6.5	0.2*M* Li_2_SO_4_	
**42**	HMW	12.0	MES, 6.5	0.1*M* KSCN	0.1*M* NaBr
**43 **	HMW	18.0	ADA, 6.5	0.2*M* ammonium sulfate	
**44**	BMW	15.0	MES, 6.2	0.15*M* CaCl_2_	5%(*v*/*v*) glycerol
**45 **	BMW	15.0	Cacodylate, 5.3	5%(*v*/*v*) Tacsimate	10%(*v*/*v*) ethylene glycol
**46**	BMW	28.0	Phosphate, 6.2	0.2*M* NaCl	
**47**	MMW	22.5	Citrate, 5.5	0.1*M* ammonium sulfate	0.05*M* Mg sulfate
**48 **	MMW	22.5	Bis-tris propane, 8.0	0.2*M* MgCl_2_, 0.01*M* CoCl_2_	10%(*v*/*v*) glycerol
**49**	LMW	25.0	MES, 6.5	0.08*M* Mg acetate	0.02*M* MgCl_2_
**50**	LMW	12.5	HEPES, 7.5	0.1*M* KCl	5%(*v*/*v*) ethylene glycol
**51 **	MMW	20.0	Tris, 7.5	0.1*M* Zn acetate	0.1*M* ZnCl_2_
**52**	MMW	20.0	PIPES, 7.0	0.1*M* MgCl_2_	0.1*M* KCl
**53**	MMW	28.0	HEPES, 7.5	0.05*M* MgSO_4_	
**54 **	HMW	15.0	HEPES, 7.5	0.1*M* Na/K phosphate	10%(*v*/*v*) ethylene glycol
**55 **	HMW	25.0	PIPES, 7.0	0.1*M* RbCl	0.1*M* Mg formate
**56**	BMW	25.0	HEPES, 7.2	0.2*M* Li_2_SO_4_	
**57 **	BMW	20.0	HEPES, 7.5	0.2*M* ammonium nitrate	
**58 **	BMW	30.0	HEPES, 7.5	0.1*M* MgCl_2_	0.1*M* RbCl
**59 **	HMW	22.5	Bis-tris propane, 7.8	0.05*M* Na citrate	0.05*M* MgCl_2_
**60 **	HMW	22.5	Bicine, 9.0	7%(*v*/*v*) Tacsimate	10%(*v*/*v*) ethylene glycol
**61**	LMW	25.0	HEPES, 7.8	0.15*M* Na citrate	
**62**	LMW	28.0	Tris, 8.5	0.2*M* NaCl	5%(*v*/*v*) glycerol
**63**	MMW	15.0	Tris, 8.0	0.15*M* NaCl	0.075*M* Na acetate
**64 **	MMW	25.0	Bis-tris propane, 8.5	0.1*M* Na formate	0.1*M* NaCl
**65 **	MMW	20.0	Bicine, 9.0	0.2*M* ammonium sulfate	0.05*M* Mg sulfate
**66 **	HMW	18.0	Bis-tris propane, 8.5	0.2*M* ammonium nitrate	10%(*v*/*v*) glycerol
**67 **	HMW	25.0	Tris, 8.0	0.2*M* MgCl_2_	0.01*M* CaCl_2_
**68**	BMW	28.0	Tris, 8.5	0.15*M* ammonium acetate	
**69 **	BMW	25.0	Bicine, 9.0	10%(*v*/*v*) 2-propanol	
**70 **	BMW	18.0	Tris, 8.0	0.2*M* ammonium sulfate	
**71 **	BMW	22.5	Bicine, 8.8	0.2*M* KCl	0.02*M* Mg sulfate
**72 **	BMW	22.5	Cacodylate, 5.5	0.1*M* Na/K tartrate	10%(*v*/*v*) ethylene glycol
**73 **	LMW	10.0	MES, 6.2	0.1*M* Mg formate	0.01*M* CoCl_2_
**74 **	LMW	25.0	Bis-tris, 6.8	0.05*M* MgCl_2_	0.15*M* Li sulfate
**75 **	MMW	25.0	HEPES, 7.5	0.2*M* ammonium sulfate	0.01*M* CdCl_2_
**76 **	MMW	18.0		0.1*M* MgCl_2_	0.1*M* KCl, 10%(*v*/*v*) ethylene glycol
**77**	MMW	12.0	MES, 6.5	0.1*M* Mg acetate	10%(*v*/*v*) ethylene glycol
**78 **	HMW	12.0	MES, 6.2	0.1*M* Mg acetate	0.1*M* KCl
**79 **	HMW	8.0	PIPES, 7.0	0.04*M* CaCl_2_	0.04*M* Na formate
**80 **	BMW	18.0	Bis-tris, 6.0	0.075*M* Na citrate	0.075*M* MgCl_2_
**81 **	BMW	15.0	Bis-tris, 6.5	0.1*M* Na acetate	0.1*M* MgCl_2_
**82**	BMW	25.0	HEPES, 7.0	0.1*M* ammonium sulfate	0.1*M* Na formate
**83 **	MMW	22.5	HEPES, 7.5	0.2*M* Na/K phosphate	10%(*v*/*v*) glycerol
**84 **	BMW	22.5	Tris, 7.5	0.3*M* NaCl	0.05*M* arginine/glutamate mix
**85**	LMW	25.0	Tris, 8.0	0.04*M* CaCl_2_	0.04*M* Na formate
**86 **	LMW	20.0	PIPES, 7.0	0.1*M* MgCl_2_	0.1*M* RbCl
**87 **	MMW	15.0	HEPES, 7.5	0.2*M* MgCl_2_	5%(*v*/*v*) 2-propanol, 10%(*v*/*v*) ethylene glycol
**88 **	MMW	12.0	HEPES, 7.0	0.15*M* Mg sulfate	0.05*M* ammonium acetate
**89 **	MMW	20.0	HEPES, 7.2	7%(*v*/*v*) Tacsimate	
**90 **	HMW	15.0	Tris, 7.2	0.1*M* ammonium acetate	0.1*M* zinc chloride
**91**	HMW	20.0	HEPES, 7.8	0.05*M* MgCl_2_	0.15*M* Li sulfate
**92**	BMW	25.0	Tris, 7.8	0.1*M* KSCN	0.1*M* NaBr
**93 **	BMW	28.0	Bis-tris propane, 8.5	0.05*M* ammonium sulfate	0.05*M* Li sulfate
**94 **	BMW	15.0	PIPES, 7.0	0.2*M* ammonium sulfate	10m*M* CdCl_2_, 10%(*v*/*v*) ethylene glycol
**95 **	BMW	22.5	Bis-tris, 7.5	0.2*M* Li_2_SO_4_	0.05*M* Zn acetate
**96**	BMW	22.5	HEPES, 7.5	0.075*M* NaBr and NaI mix	0.05*M* NaF

**Table 2:** Examples of proteins crystallized in the BCS2 screens

Proteins	Abbreviation	UniProt ID	MW (kDa)	Examples of hits	Smear precipitants	Single PEG precipitants from primary screens
Acetyl-CoA carboxylase 1 (C-terminal domain)	ACACA	Q13085	87.4	23, 65, 89, 92	MMW, BMW	3350, 5K MME
Amphiphysin (bar domain)	AMPH	P49418	24.2	2, 14, 20, 37, 39, 40, 42, 45, 66, 68, 78, 80, 96	LMW, MMW, HMW, BMW	3350
Bromodomain adjacent to zinc-finger domain protein 2B (bromodomain)	BAZ2B	Q9UIF8	13.6	3	LMW	600, 1K, 6K
ATPase family AAA domain-containing protein 2 (bromodomain)	ATAD2	Q6PL18	15.4	12, 14, 22, 31, 40, 41, 43, 44, 45, 47, 50, 54, 57, 61, 62, 63, 65, 66, 74, 78, 79, 80, 81, 92, 93	LMW, MMW, HMW, BMW	1K, 3350, 6K, 8K, 20K
Bridging integrator 2 (N-bar domain)	BIN2	Q9UBW5	28.0	3, 9, 10, 11, 15, 17, 21, 23, 26, 27, 33, 27, 33, 38, 39, 47, 49, 59, 82, 84	LMW, MMW, HMW, BMW	3350, 10K
Cyclin-dependent kinase-like 5 (kinase domain)	CDKL5	O76039	35.2	12, 18, 27, 38, 39, 48, 63, 65, 67, 83, 89	LMW, MMW, HMW	20K
DNA cross-link repair 1A protein	DCLRE1A	Q6PJP8	41.3	92	BMW	1K, 3350, 10K
5 Exonuclease apollo	DCLRE1B	Q9H816	37.8	50, 53, 61, 66	LMW, MMW, HMW	400
Dual-specificity tyrosine phosphorylation-regulated (kinase 1A kinase domain)	DYRK1A	Q13627	41.9	39, 94	MMW, BMW	300, 400, 3350
Zinc phosphodiesterase ELAC protein 1	ELAC1	Q9H777	40.7	85, 5, 80, 35, 65	LMW, MMW, BMW	3350, 4K
Mitogen-activated protein kinase 1 with inhibitor	ERK2VTX-11e complex	P28482	41.5	28, 47	MMW	
ETS translocation variant 1	ETV1DNA complex	P50549	12.4	5, 31, 39, 40, 49, 52, 53, 55, 57, 62, 68, 73, 78, 79, 81, 83, 84, 85, 89, 92, 93, 96	LMW, MMW, HMW, BMW	
Guanylate cyclase soluble subunit -1	GUCY1B3	Q02153	24.0	40, 83	MMW	1K, 3350
Glycogenin 1	GYG1	P46976	29.6	1, 4, 8, 11, 13, 14, 15, 18, 19, 22, 23, 25, 32, 33, 37, 43, 46, 52, 53, 55, 56, 57, 58, 59, 60, 67, 68, 69, 70, 71, 72, 73, 79, 80, 81, 82, 83, 84, 85, 86, 91, 92, 93, 94, 95, 96	LMW, MMW, HMW, BMW	1500, 3350, 2K MME, 8K
Kelch-like protein 2	KLHL2	O95198	30.3	9, 20, 43, 65, 66, 70, 74, 79, 80, 86, 88, 89	LMW, MMW, HMW, BMW	400, 3350, 4K, 5K MME, 6K, 8K
-Lactamase-like protein 2	LACTB2	Q53H82	32.9	21, 27, 41, 52, 59, 64, 76	MMW, HMW, BMW	2K MME, 3350, 4K, 5K MME, 6K, 10K
Methionine adenosyltransferase I	MAT1A	Q00266	43.7	33, 85	LMW, BMW	3350
Methylmalonyl-CoA epimerase, mitochondrial	MCEE	Q96PE7	14.4	3, 4, 5, 8, 10, 12, 14, 15, 20, 24, 26, 28, 32, 33, 38, 39, 40, 46, 47, 59, 60, 64, 68, 72, 82, 83, 89, 92, 96	LMW, MMW, HMW, BMW	3350, 4K, 8K, 10K
Malonyl-CoA decarboxylase, mitochondrial	MLYCD	O95822	50.4	3, 9, 22, 80, 81, 82, 92	LMW, MMW, BMW	3350, 5K MME, 6K, 8K, 10K, 20K
P300/CBP-associated factor (bromodomain)	PCAF	Q92831	14.2	11	MMW	3350, 10K
Membrane-associated tyrosine- and threonine-specific CDC2-inhibitory kinase	PKMYT1	Q99640	32.0	9, 41, 50, 62, 65, 67, 70, 76, 79, 85, 88, 89, 94	LMW, MMW, HMW, BMW	1K, 3350
Tumour protein p73-like (tetramerization domain)	TP73L	Q9H3D4	7.4	10, 87, 32, 53, 40, 19, 68, 76, 4, 25, 87, 3, 82, 54, 81, 56, 27, 26, 37	LMW, MMW, HMW, BMW	550 MME, 1K, 3350, 4K, 5K MME
GDP-L-fucose synthase	TSTA3	Q13630	35.3	3, 15, 38, 60	LMW, HMW	3350, 4K, 6K, 10K
MAP kinase 14 complexed with TAB1-activating peptide	p38TAB1 complex	Q16539	44.4	9, 75	MMW	3350
Cyclin-G-associated kinase (kinase domain)	GAKnanobody complex	O14976	53.8	15, 22	HMW, BMW	3350, 5K MME, 10K
RAS-association domain-containing protein 3	RASSF3	Q86WH2	16.2	47	MMW	4000
Complex of SARAH domains from RASSF5 and MST2	RASSF5MST2 SARAH	Q8WWW0 and Q13188	12.3	5, 6, 11, 12, 18, 24, 36, 44, 48, 52, 60, 62, 64, 67, 71, 74, 76, 84, 87, 92, 93	LMW, MMW, HMW, BMW	3350
Bloom syndrome protein	BLMnanobody complex	P54132	92.1	79	HMW	20K
Cullin-3 and Kelch-like protein 11	CUL3KLHL11 complex	Q13618 and Q9NVR0	77.3	10, 15, 22, 43, 56, 68, 70, 74	LMW, MMW, HMW, BMW	3350, 10K
Bromodomain-containing protein 9 (bromodomain)	BRD9	Q9H8M2	14.2	5, 11, 34, 45, 90	LMW, MMW, HMW, BMW	1K, 2K MME, 3350, 6K, 8K
Bromodomain and WD repeat-containing protein 1 (bromodomain)	WDR9	Q9NSI6	14.4	4, 5	LMW	3350
